# Deaths burden of laryngeal cancer attributable to occupational exposure in the male working age population: findings from global burden of disease study 2021

**DOI:** 10.3389/fpubh.2025.1605654

**Published:** 2025-06-27

**Authors:** Donghai Wang, Sen Yang, Wenxing Yu, Zixuan Tang, Qin Wang, Mingzhu Fang, Chunyan Li

**Affiliations:** ^1^Department of Otolaryngology-Head and Neck Surgery, Suining Central Hospital, Suining, China; ^2^Department of Ophthalmology, Suining Central Hospital, Suining, China; ^3^Department of Neurology, Sichuan Provincial People’s Hospital Medical Group Chuantou Xichang Hospital, Xichang, China

**Keywords:** occupational exposure, laryngeal cancer, global burden of disease, working age population, epidemiology

## Abstract

**Objectives:**

Laryngeal cancer (LC) is a major health threat to the male working-age population, with occupational exposures (asbestos and sulfuric acid) being key risk factors. However, the deaths burden of LC attributable to occupational exposure in this population remains unclear. This study analyzes the deaths burden from 1990 to 2021 using Global Burden of Disease (GBD) data, providing insights for optimizing prevention strategies.

**Methods:**

Data from GBD 2021 database were used to calculate age-standardized death rates (ASDR) and estimated annual percent changes (EAPC). Decomposition analysis and Bayesian age-period-cohort (BAPC) models were applied to assess trends, drivers, and predict future trends to 2040. Frontier analysis exploring the relationship between SDI and ASDR for 204 countries and territories.

**Results:**

Globally, deaths burden of LC attributable to occupational exposures exhibited divergent trends from 1990 to 2021. Asbestos-related deaths decreased by 22% (533.6 cases in 2021; ASDR 0.02/100,000, 95% CI 0.01–0.04), with an EAPC of −3.23% (95% CI -3.32 to −3.14). Sulfuric acid-associated mortality increased by 16.19% (2,131.2 cases; ASDR 0.09/100,000, 95% CI 0.04–0.16), though with an EAPC decline of −1.90% (−1.98 to −1.82), suggesting improving age-standardized rates. Regional disparities were striking: High-SDI regions showed elevated asbestos burdens (Western Europe ASDR 0.06 vs. global 0.02), while middle-SDI regions dominated sulfuric acid mortality (South Asia ASDR 0.18 vs. global 0.09). Decomposition analysis revealed distinct drivers – asbestos burden growth (415% from epidemiological factors) reflected latency effects of historical exposures, whereas sulfuric acid increases stemmed from population expansion (336%) and aging (148%). Projections to 2040 indicate continued divergence: asbestos-related deaths are predicted to decline to 372.8 cases (ASDR 0.011/100,000), while sulfuric acid-associated mortality may rise to 2,543.8 cases (ASDR 0.078/100,000), with middle/low-SDI regions accounting for 78% of sulfuric acid burden.

**Conclusion:**

Laryngeal cancer burden attributable to occupational exposure shows a contrasting trend with asbestos-related deaths appearing to decrease while sulfuric acid-related cases are rising. High-SDI regions face legacy asbestos risks, while middle and low-SDI regions are increasingly exposed to sulfuric acid. Targeted prevention strategies are needed, high-SDI regions should focus on asbestos risk management, and middle/low-SDI regions need to strengthen industrial protections, promote alternative materials, and enhance screening.

## Introduction

Laryngeal cancer (LC) is a prevalent malignant tumor of the head and neck, primarily originating from the epithelial cells of the larynx (1). Its development severely impacts patients’ ability to speak, breathe, and swallow, with most cases diagnosed at advanced stages, leading to high disability and mortality rates (2). This has long posed a significant challenge to global public health. Despite advancements in medical technology and public health measures, the 5-year survival rate for LC patients remains below 60% (3). Data shows that in 2020, there were 184,615 newly diagnosed LC cases worldwide, and the death toll was as high as 99,840 (4). Therefore, the development of LC represents a substantial clinical, health, and economic burden.

Studies have shown that LC burden in working-age people has been increasing, and the incidence of LC in males is about 7 times that of females (5). Male patients of working age are usually at the peak of their productive capacity, and the diagnosis of LC not only reduces the labor force participation rate, but also brings a significant socio-economic burden to the family and society (6). Therefore, the high incidence of LC in working-age men has attracted the attention of scholars around the world. However, current research on the burden of LC has mainly focused on all-age populations or individual behavioral risk factors such as smoking and excessive alcohol consumption (7). Studies on the global distribution and characteristics of LC caused by occupational exposure in working-age men are insufficient.

Occupational exposure refers to the contact with harmful physical, chemical or biological agents in the workplace environment (8). For LC, occupational carcinogens include asbestos, sulfuric acid, diesel exhaust gas and nickel compounds. Studies on the global burden of LC show that asbestos and sulfuric acid are the two major attributable risk factors after smoking and drinking (9). Although occupational exposure is an intervenable risk factor and certain research progress has been made, its early diagnosis and treatment still face many challenges. Especially in most parts of Asia, Africa and some countries in Latin America, asbestos and sulfuric acid are still widely used, and the risk of occupational exposure in major asbestos-consuming countries such as China and India is still on the rise (10). Although high-income countries have occupational exposure limits, the historical burden of diseases still exists, resulting in an unbalanced regional distribution of the burden of LC globally.

Understanding the epidemiology and trends of LC attributable to occupational exposure across different regions and countries is essential for effective prevention, early detection, and treatment. Thus, we used Global Burden of Disease (GBD) 2021 data to conduct a comprehensive analysis of the trends in the death burden of LC attributable to occupational exposure in the male working-age population since 1990, at global, regional, and national levels. Age-standardized rates (ASR) were used to assess the deaths burden and its temporal trends. Decomposition analysis were employed to quantify the main driving factors of death cases. Additionally, frontier analysis identified the potential for improvement in countries with different Sociodemographic Index Index (SDI) levels. Furthermore, we projected the burden trends through 2040. To fill a critical gap in the global distribution map of occupational LC and provides valuable insights for optimizing occupational health resource allocation and developing more targeted global prevention strategies.

## Methods

### Data acquisition

This study is derived from the GBD 2021 dataset, a globally recognized epidemiological research platform that integrates multidimensional data from 204 countries and territories covering 371 diseases and health issues, and 88 risk factors between 1990 and 2021 (11). The GBD research network standardizes the data through cleaning, correction, and modeling processes to ensure comparability and reliability. The definition of LC is based on the International Classification of Diseases (ICD) coding system, primarily including C32-C32.9 in ICD-10 and corresponding codes 161–161.9, V10.21 in ICD-9. The database, built with the collaborative efforts of over 11,500 researchers, offers a comprehensive assessment system. For research on LC attributable to occupational exposure, mortality data was extracted from the Global Health Data Exchange (GHDx^1^) and analyzed by factors such as age, gender (male working-age population in 15 to 64 years), and geographic distribution. To further investigate the impact of socio-economic factors on deaths burden, the study incorporated the SDI framework^2^. The SDI, which is based on per capita income, education level, and fertility rate, divides regions into five development tiers, ranging from low to high.

### Estimation of deaths burden

To better understand the distribution characteristics of deaths burden and its influencing factors, the research explores demographic and socio-economic factors, with a particular focus on the impact of population structure and the SDI on the variation in deaths burden across different regions. In terms of trend analysis, a longitudinal research design was employed, using long-term data from 1990 to 2021. The study evaluates temporal trends in deaths burden by calculating the estimated annual percentage change (EAPC). Which is calculated based on a logarithmic linear regression model, with the core formula being: ln(ASR) = a + bx + e, where ln(ASR) represents the natural logarithm of the ASR, x is the year variable, a is the model intercept, b is the regression coefficient, and e is the error term. The EAPC is calculated from the regression coefficient b using the formula: EAPC = (exp(b) – 1) × 100. To assess the statistical significance of trend changes, the 95% confidence interval (CI) was calculated. A CI upper bound below 0 indicates a significant decreasing trend in deaths burden, while a lower bound above 0 suggests a significant increasing trend (12).

### Decomposition analysis

This study employs a decomposition analysis method to investigate the driving factors in the deaths cases of LC attributable to occupational exposure. It systematically evaluates the impact mechanisms of population structure changes, population size expansion, and the evolution of epidemiological characteristics on deaths burden from 1990 to 2022. Following Das Gupta’s decomposition framework, changes in deaths (ΔD) were partitioned into three components: ΔD=D2021 − D1990 = Δage+Δpop+Δepi, where Δage (aging), Δpop (population growth), and Δepi (epidemiological rate changes) were calculated via counterfactual scenarios (13). Notably, changes in epidemiological characteristics, including improved access to healthcare and the widespread adoption of preventive measures, have had complex effects on deaths burden. Through quantitative analysis, this study identifies the contributions of each factor to the deaths cases of LC, providing a scientific basis for a comprehensive understanding of the epidemiological evolution.

### Predictive analysis

This study employs the Bayesian Age-Period-Cohort (BAPC) model to predict the evolving trends in the deaths burden of LC attributable to occupational exposure. The prediction model, implemented on the R language platform using the Integrated Nested Laplace Approximation (INLA, 23.09.09) algorithm and the BAPC (0.0.36) package, is capable of effectively handling complex hierarchical data structures. The model not only captures the disease risk characteristics across different age groups but also reflects the influence of social environmental changes and medical advancements on disease patterns (14). Compared to traditional prediction methods, the BAPC model offers superior explanatory power and predictive accuracy, particularly in long-term trend analysis and intergenerational comparisons. This makes it a reliable analytical tool for gaining deeper insights into the epidemiological characteristics of LC attributable to occupational exposure. Using this modeling framework, we forecast mortality rates from 2022 to 2040, which will provide crucial evidence for optimizing health resource allocation and formulating disease prevention strategies.

### Frontier analysis

This study employs an advanced analytical approach to construct an evaluation model based on ASDR, aimed at exploring the association between the deaths burden and the SDI. Compared to traditional linear regression models, the frontier analysis method offers significant advantages: it not only identifies multidimensional driving factors influencing deaths burden but also effectively captures the complex non-linear relationship between SDI and deaths burden. Methodologically, this study introduces an innovative approach by combining Locally Weighted Regression (LOESS) with Local Polynomial Regression, using a smoothing span parameter of 0.3 to construct a non-linear boundary curve between SDI and ASDR. This boundary curve holds substantial theoretical significance, as it establishes the achievable theoretical minimum ASDR for each country or region at its specific development level, providing a scientific benchmark for evaluating national disease prevention and control performance. Additionally, by quantifying the gap between the observed disease burden and the theoretical minimum value, this method precisely identifies areas that require focused improvement (15). In order to validate the robustness and reliability of the results, we performed 1,000 bootstrap resamples, calculating the average ASDR for each SDI value to effectively control for data fluctuations. In the evaluation phase, we constructed a quantifiable assessment indicator system by measuring the absolute distance between each country’s actual ASDR in 2021 and the boundary curve. This indicator not only reflects the current effectiveness of national disease control but also evaluates the potential for future reductions in the LC attributable to occupational exposure burden.

### Statistics analysis

This study uses standardized epidemiological indicators to quantify the deaths burden, with ASDR calculated per 100,000 person-years and reported alongside their 95% Confidence Interval (CI) to reflect the precision of the predictions. To ensure the robustness of the findings, multiple statistical models and techniques were applied to analyze temporal trends, decompose contributing factors, project future patterns, and evaluate disparities across sociodemographic levels. Each model was selected based on its suitability for the corresponding research objective, and all outputs were visually represented through standardized plots and comparative graphs. Additionally, the EAPC is also provided with a 95% CI. The entire data analysis and visualization process was performed using R Studio (Version 4.3.3 for Windows). For statistical inference, we used two-tailed tests, with a *p*-value < 0.05 indicating significance.

## Results

### Global level

**Figure 1:**
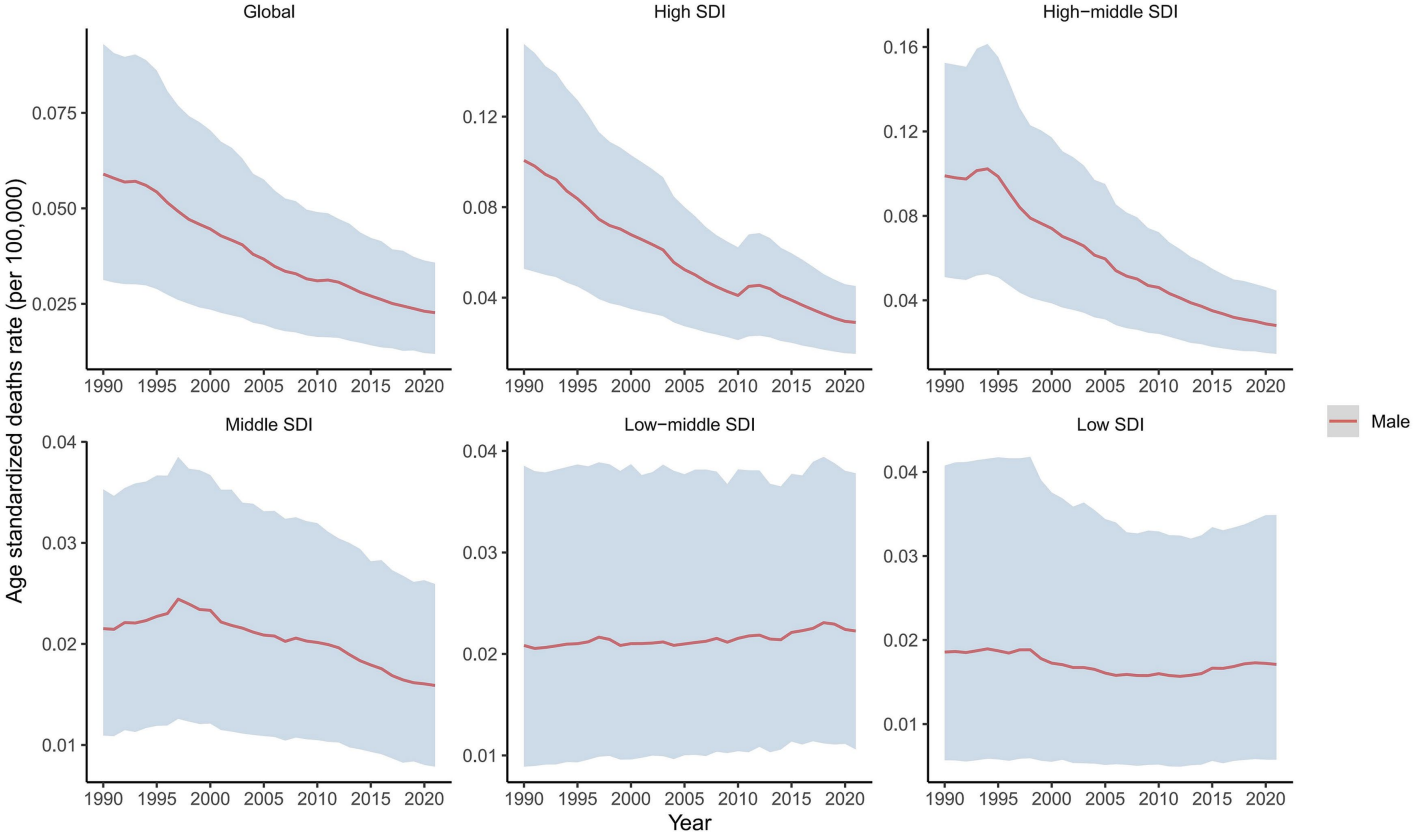
Temporal trend in ASDRs of laryngeal cancer attributable to occupational exposure to asbestos, globally and by SDI, 1990–2021.

**Figure 2:**
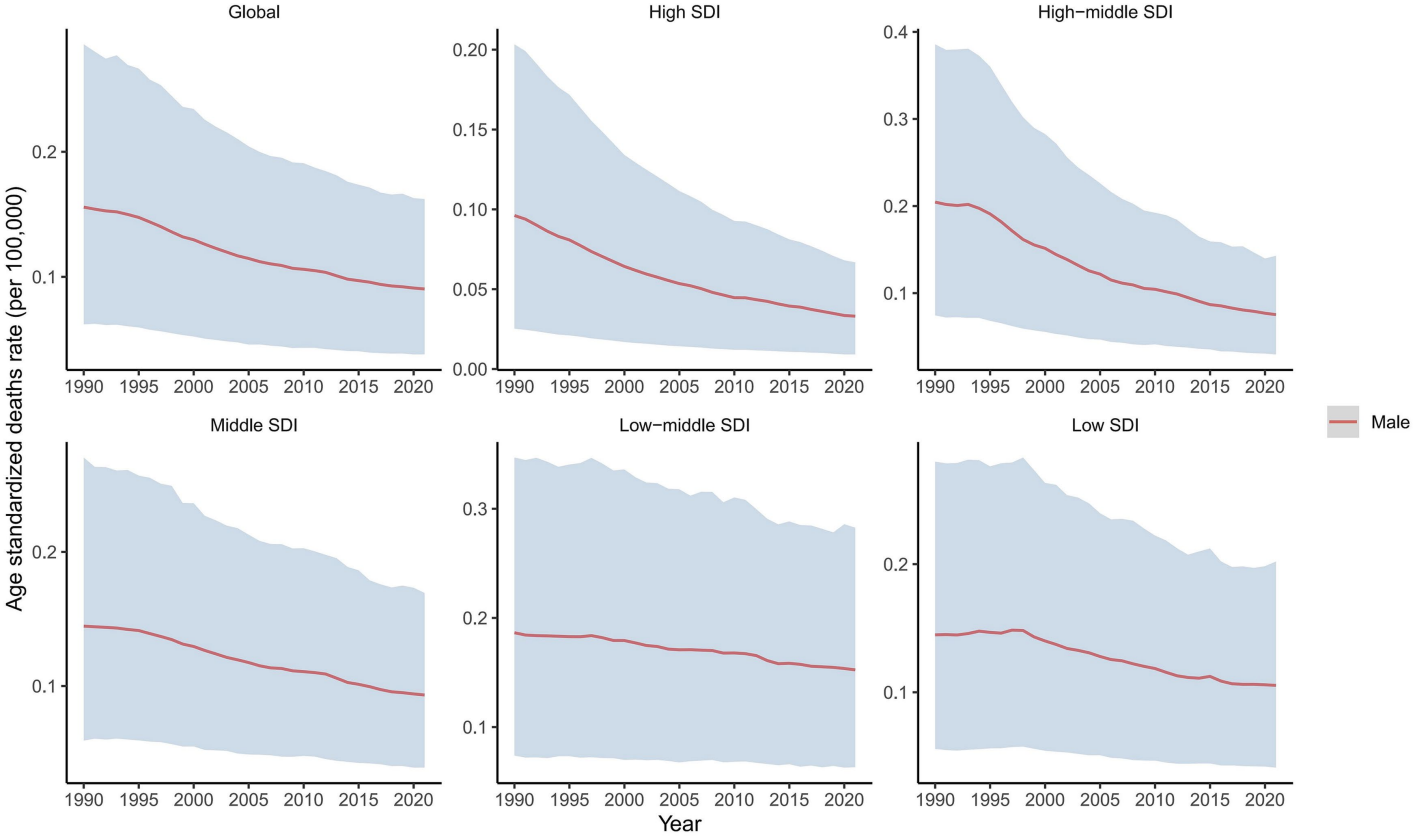
Temporal trends in ASDRs of laryngeal cancer from occupational sulfuric acid exposure, globally and by SDI, 1990–2021.

In 2021, the global male LC attributable to occupational exposure to asbestos resulted in a total of 533.63 deaths (95% CI: 280.15, 842.41), representing a 22% decrease compared to 1990. The ASDR was 0.02 per 100,000, with an average annual decrease of −3.23% since 1990 (Table 1 and Figure 1). For LC attributable to occupational exposure to sulfuric acid, the total number of male deaths in 2021 was 2131.15 (95% CI: 899.19, 3825.72), showing a 16.19% increase compared to 1990. The ASDR was 0.09 per 100,000, with an average annual decrease of −1.90% (Table 2 and Figure 2). Across different SDI regions, the High-middle SDI region had the highest number of male deaths cases from LC attributable to occupational exposure to asbestos in 2021, with 147.25 deaths (95% CI: 76.86, 234.07), while the Low SDI region had the lowest with 26.24 deaths (95% CI: 8.72, 53.90). The highest ASDRs were observed in the High and High-middle SDI regions, while the lowest were in the other SDI regions. The highest annual increase in ASDR was observed in the Low-middle SDI region (0.27%), and the fastest decrease was in the High-middle SDI region (−4.49%) (Table 1 and Figure 1A). For male deaths from LC attributable to occupational exposure to sulfuric acid in 2021, the highest number was in the Middle SDI region (741.14), and the lowest was in the High SDI region (148.37). The highest ASDR was found in the Low-middle SDI region (0.15 per 100,000), and the lowest was in the High SDI region (0.03 per 100,000). The fastest annual decrease in ASDR was in the High-middle SDI region (−3.48%), while the slowest decrease occurred in the Low-middle SDI region (−0.68%) (Table 2 and Figure 2).

**Table 1:** ASDR of LC attributable to occupational exposure to asbesto (1990–2021) and EAPC by region.

Group	1990		2021		1990–2021
	Deaths cases, (95% CI)	ASDRs per 100,000 (95% CI)	Deaths cases, (95% CI)	ASDRs per 100,000 (95% CI)	EAPC, %, (95% CI)
Global	684.46(362.88,1079.96)	0.06(0.03,0.09)	533.63(280.15,842.41)	0.02(0.01,0.04)	−3.23(−3.32,−3.14)
SDI					
High	271.18(142.09,409.59)	0.10(0.05,0.15)	137.12(71.68,212.81)	0.03(0.02,0.05)	−3.92(−4.10,−3.74)
High-middle	284.67(146.75,438.39)	0.10(0.05,0.15)	147.25(76.86,234.07)	0.03(0.01,0.04)	−4.49(−4.66,−4.31)
Middle	72.49(36.88,118.91)	0.02(0.01,0.04)	127.45(63.01,207.86)	0.02(0.01,0.03)	−1.17(−1.39,−0.95)
Low-middle	41.49(17.66,76.90)	0.02(0.01,0.04)	94.71(45.02,160.95)	0.02(0.01,0.04)	0.27(0.21,0.34)
Low	13.73(4.17,30.23)	0.02(0.01,0.04)	26.24(8.72,53.90)	0.02(0.01,0.03)	−0.46(−0.66,−0.27)
Regions					
Andean Latin America	0.61(0.24,1.22)	0.01(0.00,0.02)	0.70(0.24,1.56)	0.00(0.00,0.01)	−3.42(−3.95,−2.89)
Australasia	11.06(5.55,17.56)	0.19(0.09,0.30)	3.47(1.75,5.70)	0.03(0.01,0.05)	−6.32(−6.55,−6.09)
Caribbean	2.26(1.08,3.86)	0.03(0.02,0.06)	4.56(1.90,8.74)	0.03(0.01,0.06)	−0.05(−0.17,0.07)
Central Asia	7.01(3.61,11.27)	0.05(0.03,0.08)	5.37(2.55,9.14)	0.02(0.01,0.03)	−3.28(−3.57,−2.98)
Central Europe	32.74(16.99,53.03)	0.08(0.04,0.13)	36.80(18.62,59.64)	0.07(0.04,0.12)	0.29(0.01,0.57)
Central Latin America	4.47(2.30,6.97)	0.02(0.01,0.03)	7.21(3.36,12.05)	0.01(0.00,0.02)	−2.12(−2.32,−1.92)
Central Sub-Saharan Africa	1.09(0.12,3.36)	0.02(0.00,0.05)	2.50(0.32,7.74)	0.01(0.00,0.04)	−0.48(−0.77,−0.19)
East Asia	24.17(10.58,42.86)	0.01(0.00,0.01)	36.45(14.91,67.65)	0.01(0.00,0.01)	−0.66(−1.06,−0.26)
Eastern Europe	94.41(47.88,147.79)	0.13(0.07,0.20)	50.46(24.96,82.51)	0.06(0.03,0.10)	−3.45(−3.90,−3.01)
Eastern Sub-Saharan Africa	3.07(0.37,9.26)	0.01(0.00,0.04)	5.83(0.82,17.05)	0.01(0.00,0.03)	−0.80(−0.89,−0.70)
High-income Asia Pacific	7.46(3.83,11.88)	0.01(0.01,0.02)	2.93(1.47,4.87)	0.00(0.00,0.01)	−3.86(−4.46,−3.27)
High-income North America	49.57(25.55,78.12)	0.06(0.03,0.10)	27.03(14.11,43.14)	0.02(0.01,0.03)	−5.28(−5.65,−4.90)
North Africa and Middle East	33.49(11.93,71.34)	0.06(0.02,0.13)	25.26(10.26,48.68)	0.02(0.01,0.03)	−4.39(−4.75,−4.02)
Oceania	0.04(0.01,0.08)	0.00(0.00,0.01)	0.09(0.03,0.19)	0.00(0.00,0.01)	0.23(−0.04,0.49)
South Asia	49.08(19.59,92.42)	0.02(0.01,0.05)	106.06(45.85,183.66)	0.02(0.01,0.04)	−0.24(−0.42,−0.07)
Southeast Asia	9.37(4.35,16.57)	0.01(0.01,0.02)	21.60(9.74,38.82)	0.01(0.00,0.02)	−0.56(−0.66,−0.46)
Southern Latin America	10.93(5.17,18.21)	0.09(0.04,0.15)	8.14(4.03,13.81)	0.04(0.02,0.07)	−2.52(−3.00,−2.03)
Southern Sub-Saharan Africa	9.39(4.15,16.87)	0.13(0.06,0.23)	18.54(8.98,30.71)	0.12(0.06,0.19)	−1.07(−2.15,0.01)
Tropical Latin America	26.76(13.58,42.29)	0.10(0.05,0.16)	48.94(25.15,77.50)	0.07(0.04,0.11)	−1.06(−1.22,−0.90)
Western Europe	302.31(161.93,456.44)	0.22(0.12,0.34)	113.38(59.47,173.16)	0.06(0.03,0.09)	−4.15(−4.34,−3.95)
Western Sub-Saharan Africa	5.18(1.88,10.45)	0.02(0.01,0.04)	8.30(3.12,16.52)	0.01(0.01,0.03)	−0.74(−0.91,−0.58)

ASDR, age standardized deaths rate; LC, laryngeal cancer; EAPC, estimated annual percentage change; SDI, socio-demographic index; 95% UI, 95% uncertainty interval; 95% CI, 95% confidence interval.

**Table 2:** ASDR of LC attributable to occupational exposure to sulfuric acid exposure (1990–2021) and EAPC by region.

Group	1990		2021		1990–2021
	Deaths cases, (95% CI)	ASDRs per 100,000 (95% CI)	Deaths cases, (95% CI)	ASDRs per 100,000 (95% CI)	EAPC, %, (95% CI)
Global	1834.13(731.48,3365.65)	0.16(0.06,0.29)	2131.15(899.19,3825.72)	0.09(0.04,0.16)	−1.90(−1.98,−1.82)
SDI					
High	254.16(66.84,537.56)	0.10(0.03,0.20)	148.37(40.72,299.68)	0.03(0.01,0.07)	−3.43(−3.53,−3.32)
High-middle	580.28(211.33,1094.17)	0.20(0.07,0.39)	389.23(153.26,738.43)	0.08(0.03,0.14)	−3.48(−3.60,−3.35)
Middle	498.19(204.26,930.18)	0.14(0.06,0.27)	741.14(311.41,1344.96)	0.09(0.04,0.17)	−1.54(−1.59,−1.49)
Low-middle	386.92(153.54,720.48)	0.19(0.07,0.35)	671.79(278.92,1246.57)	0.15(0.06,0.28)	−0.68(−0.72,−0.63)
Low	112.17(43.18,216.82)	0.14(0.06,0.28)	178.11(69.67,341.91)	0.11(0.04,0.20)	−1.30(−1.40,−1.19)
Regions					
Andean Latin America	3.72(1.51,7.21)	0.06(0.02,0.12)	5.73(2.29,10.85)	0.04(0.01,0.07)	−1.95(−2.16,−1.75)
Australasia	3.79(0.88,8.45)	0.07(0.02,0.15)	1.93(0.45,4.36)	0.02(0.00,0.04)	−4.16(−4.37,−3.96)
Caribbean	12.62(5.15,23.33)	0.18(0.07,0.33)	30.95(12.75,59.23)	0.21(0.09,0.41)	0.82(0.69,0.94)
Central Asia	42.35(17.43,76.58)	0.30(0.12,0.54)	22.49(9.27,41.70)	0.09(0.04,0.16)	−4.22(−4.38,−4.05)
Central Europe	102.90(24.23,226.20)	0.26(0.06,0.58)	67.42(15.88,144.28)	0.14(0.03,0.31)	−2.10(−2.21,−1.99)
Central Latin America	30.75(13.00,55.31)	0.12(0.05,0.22)	41.10(16.88,73.50)	0.06(0.02,0.11)	−2.85(−3.00,−2.71)
Central Sub-Saharan Africa	7.28(2.55,14.87)	0.10(0.03,0.20)	12.78(4.74,25.52)	0.06(0.02,0.13)	−1.52(−1.66,−1.39)
East Asia	317.61(125.48,615.83)	0.11(0.04,0.21)	385.77(154.00,757.85)	0.06(0.02,0.12)	−1.94(−2.05,−1.83)
Eastern Europe	205.83(49.37,448.59)	0.29(0.07,0.63)	72.74(18.12,161.78)	0.09(0.02,0.21)	−4.13(−4.34,−3.91)
Eastern Sub-Saharan Africa	24.94(9.66,47.04)	0.10(0.04,0.19)	50.37(18.65,98.69)	0.09(0.03,0.17)	−0.73(−0.83,−0.63)
High-income Asia Pacific	22.96(5.48,51.14)	0.04(0.01,0.09)	7.28(1.68,16.05)	0.01(0.00,0.02)	−5.19(−5.34,−5.04)
High-income North America	49.37(12.00,108.38)	0.07(0.02,0.14)	44.28(10.82,96.12)	0.03(0.01,0.06)	−2.77(−2.87,−2.68)
North Africa and Middle East	86.14(35.07,161.79)	0.15(0.06,0.28)	119.60(49.37,218.56)	0.08(0.03,0.14)	−2.23(−2.32,−2.15)
Oceania	0.19(0.07,0.39)	0.02(0.01,0.04)	0.42(0.16,0.88)	0.01(0.01,0.03)	−0.45(−0.50,−0.40)
South Asia	513.15(202.82,951.72)	0.25(0.10,0.46)	829.77(345.85,1558.19)	0.18(0.08,0.34)	−1.13(−1.20,−1.05)
Southeast Asia	69.07(27.74,127.35)	0.08(0.03,0.16)	168.27(69.49,313.20)	0.08(0.03,0.15)	−0.08(−0.15,−0.00)
Southern Latin America	40.30(16.36,74.86)	0.33(0.14,0.62)	19.67(7.85,36.37)	0.09(0.04,0.17)	−3.96(−4.08,−3.85)
Southern Sub-Saharan Africa	11.03(4.34,20.96)	0.14(0.06,0.27)	9.71(3.74,18.82)	0.06(0.02,0.11)	−3.19(−3.68,−2.69)
Tropical Latin America	72.09(29.37,131.61)	0.26(0.11,0.47)	125.23(50.88,229.59)	0.18(0.07,0.33)	−1.18(−1.37,−0.98)
Western Europe	197.99(48.59,432.00)	0.15(0.04,0.34)	76.91(18.48,167.93)	0.04(0.01,0.09)	−4.22(−4.33,−4.11)
Western Sub-Saharan Africa	20.07(7.92,38.61)	0.07(0.03,0.13)	38.74(15.75,75.42)	0.06(0.02,0.12)	−0.28(−0.32,−0.23)

ASDR, age standardized deaths rate; LC, laryngeal cancer; EAPC, estimated annual percentage change; SDI, socio-demographic index; 95% UI, 95% uncertainty interval; 95% CI, 95% confidence interval.

### Regional level

**Figure 3:**
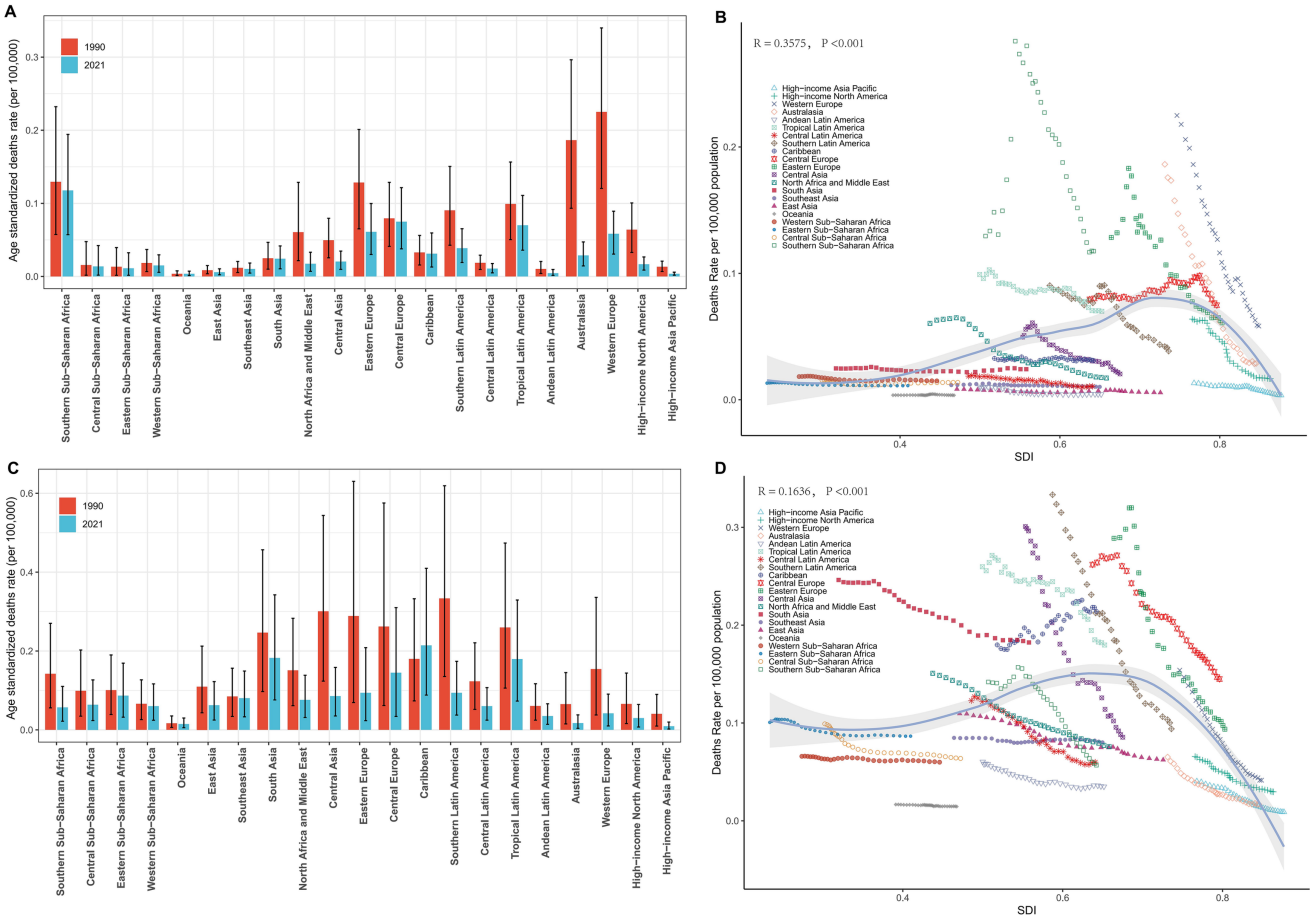
Regional ASDRs of laryngeal cancer from occupational exposure, 1990–2021. **(A,B)** Asbestos-related ASDRs across 21 regions and by SDI. **(C,D)** Sulfuric acid-related ASDRs across 21 regions and by SDI.

In 2021, an analysis of 21 GBD regions revealed significant regional differences in male LC attributable to occupational exposure. For occupational exposure to asbestos, the highest ASDR were reported in Southern Sub-Saharan Africa (0.12), Central Europe (0.07), and Tropical Latin America (0.07), while Oceania, High-income Asia Pacific, and Andean Latin America reported the lowest ASDRs (Table 1). From 1990 to 2021, Central Europe (0.29%) and Oceania (0.23%) exhibited an increasing trend in ASDR, while Australasia (−6.32%), High-income North America (−5.28%), and North Africa and the Middle East (−4.39%) experienced the most significant annual percentage decreases in ASDR (Table 1). Additionally, the comparison of differences in ASDR for LC attributable to occupational exposure to asbestos across different years in the 21 regions is shown in Figure 3A.

Similarly, in 2021, for male deaths from LC attributable to occupational exposure to sulfuric acid, the highest ASDRs were reported in the Caribbean (0.21), South Asia (0.18), and Tropical Latin America (0.18), while the lowest ASDRs were found in High-income Asia Pacific (0.01), Oceania (0.01), and Australasia (0.02) (Table 2). From 1990 to 2021, the Caribbean (0.82%) showed an increasing trend in ASDR, while High-income Asia Pacific (−5.19%), Western Europe (−4.22%), and Central Asia (−4.22%) exhibited the most significant decreases in annual percentage change (EAPC) (Table 2). The comparison of differences in male LC attributable to occupational exposure to sulfuric acid deaths across different years in the 21 regions is shown in Figure 3C.

At the regional level, we found a general positive correlation between SDI and the ASDR for LC attributable to occupational exposure to asbestos from 1990 to 2021. As SDI increased, the ASDR for LC showed an upward trend (Figure 3B). From 1990 to 2021, the ASDR in the Southern Sub-Saharan Africa region was significantly higher than its expected value, while the ASDR in Oceania was notably lower than expected during the same period (Figure 3B). A similar positive correlation was observed for LC attributable to occupational exposure to sulfuric acid (Figure 3D).

### National level

**Figure 4:**
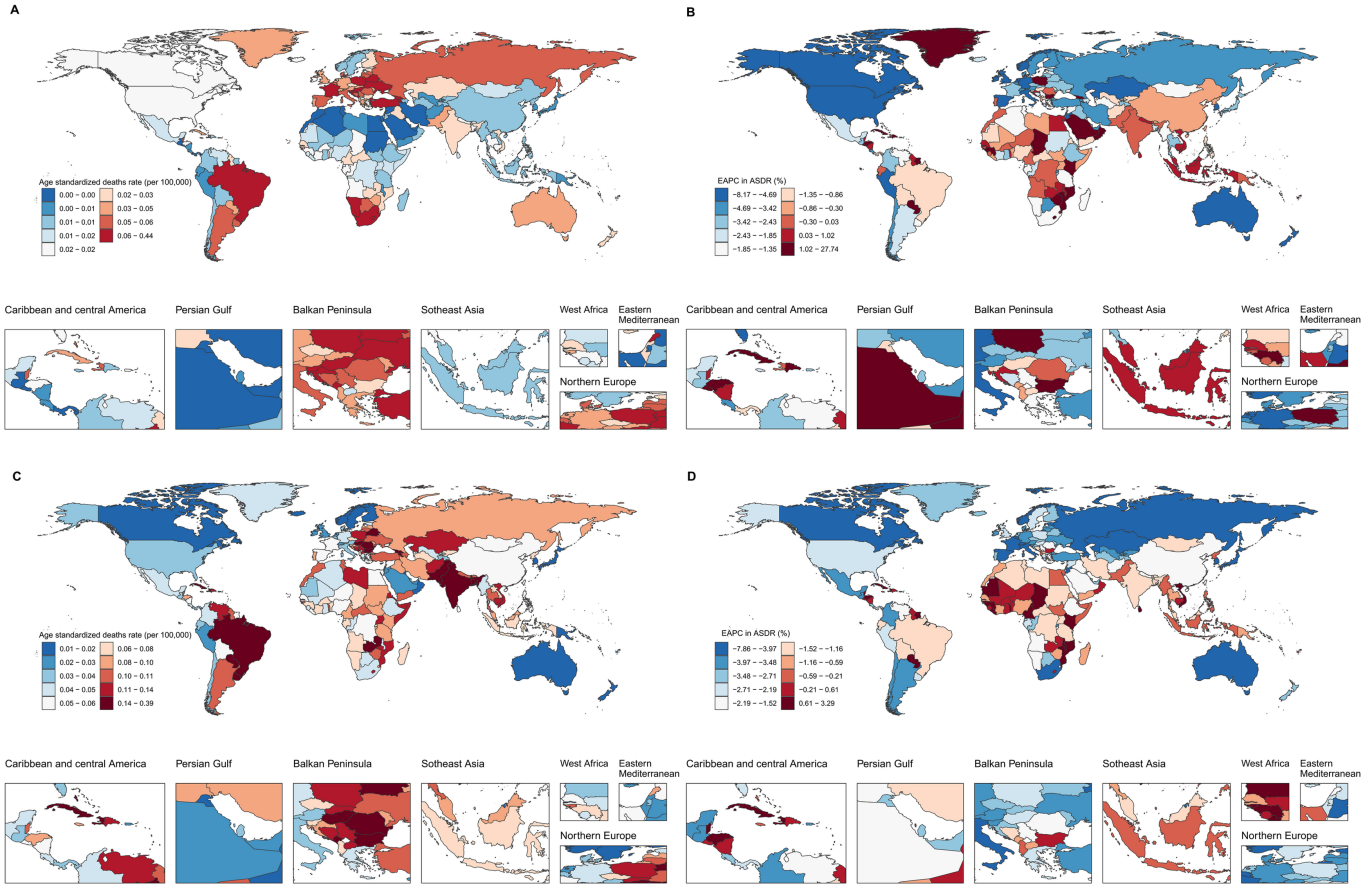
Global ASDRs and annual change in laryngeal cancer deaths from occupational exposure, 2021 and 1990–2021. **(A,B)** Asbestos. **(C,D)** Sulfuric acid.

In 2021, the ASDR for LC attributable to occupational exposure to asbestos varied significantly across countries, ranging from 0 to 0.43 per 100,000 population. Lesotho reported the highest ASDR (0.43 per 100,000), followed by Monaco (0.20) and Eswatini (0.19) (Figure 4A). Between 1990 and 2021, the countries with the fastest average annual increases in ASDR were Saudi Arabia (27.47%), Georgia (12.98%), and El Salvador (9.57%). Conversely, the most significant average annual decreases were observed in Singapore (−8.09%), American Samoa (−7.81%), and the Republic of Korea (−6.97%) (Figure 4B).

For LC attributable to occupational exposure to sulfuric acid, the ASDR ranged from 0.01 to 0.38 per 100,000 in 2021. Cuba had the highest rate (0.38), followed by Pakistan (0.31) and Seychelles (0.25) (Figure 4C). From 1990 to 2021, the countries with the most rapid increases in ASDR included Solomon Islands (3.26%), Kiribati (2.82%), and Sri Lanka (2.64%), while the largest declines occurred in the Republic of Korea (−7.78%), Singapore (−6.10%), and Spain (−5.56%) (Figure 4D).

**Figure 5:**
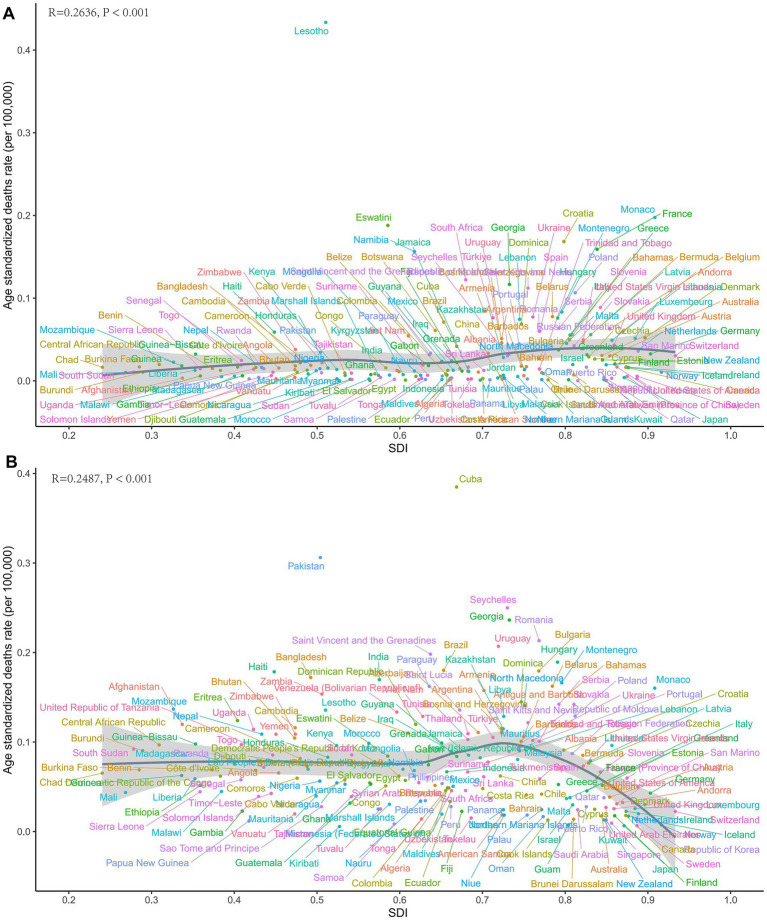
Country-level ASDRs of laryngeal cancer from occupational exposure in 2021. **(A)** Asbestos. **(B)** Sulfuric acid.

At the national level, in 2021, ASDR for LC attributable to asbestos exposure showed a positive correlation with SDI. Notably, Lesotho’s deaths burden was significantly higher than expected for its development level (Figure 5A). Similarly, for LC attributable to occupational exposure to sulfuric acid, Cuba’s burden was also markedly above the expected value based on its SDI (Figure 5B).

### Decomposition analysis

**Figure 6:**
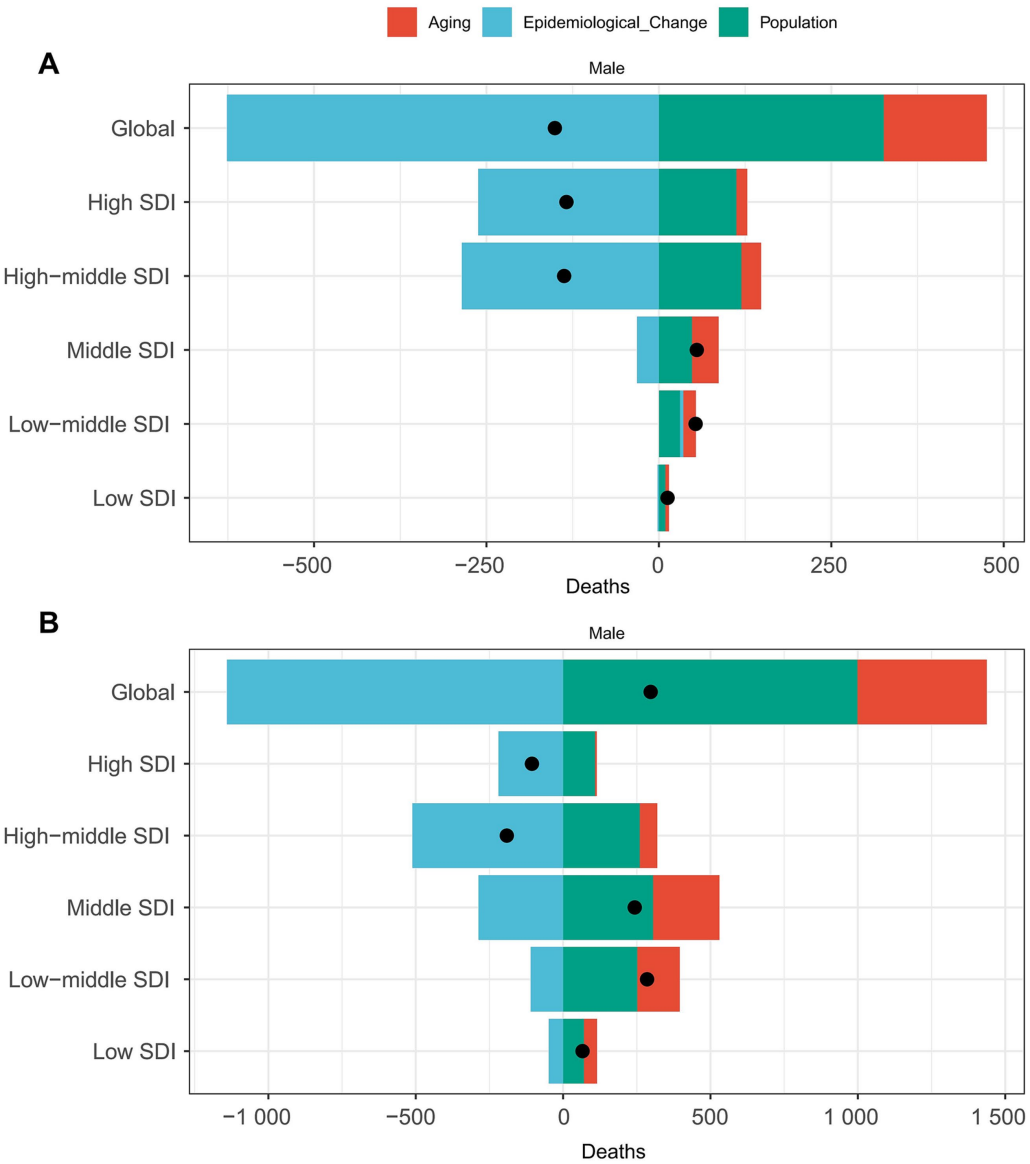
Decomposition of laryngeal cancer deaths from occupational exposure by SDI, 2021. **(A)** Asbestos. **(B)** Sulfuric acid.

To explore the driving factors behind the changes in the deaths burden of LC attributable to occupational exposure, we assessed the effects of aging, population growth, and epidemiological changes over the past 32 years. Specifically, between 1990 and 2021, the global increase in LC attributable to occupational exposure to asbestos deaths was primarily driven by epidemiological changes (415.13%), while population growth (−216.16%) and aging (−98.96%) had offsetting effects (Figure 6A). In different SDI regions, aging was the largest driver of death cases in the middle SDI region (70.24%), while it had the greatest offsetting effect in the high-middle SDI region (−20.96%). Population growth was the primary driver of death cases in the middle SDI region (87.21%) and had the largest offsetting effect in the high-middle SDI region (−86.95%).

However, for LC attributable to occupational exposure to sulfuric acid, epidemiological changes played a protective role (−383.85%), while population growth (335.99%) and aging (147.86%) were the primary drivers of increased death cases (Figure 6B). In different SDI regions, aging was the largest driver of death case growth in the middle SDI region (92.78%), population growth was the main driver in the middle SDI region (125.47%), and epidemiological factors had the largest impact in the high SDI region (207.35%).

### Predictive analysis

**Figure 7:**
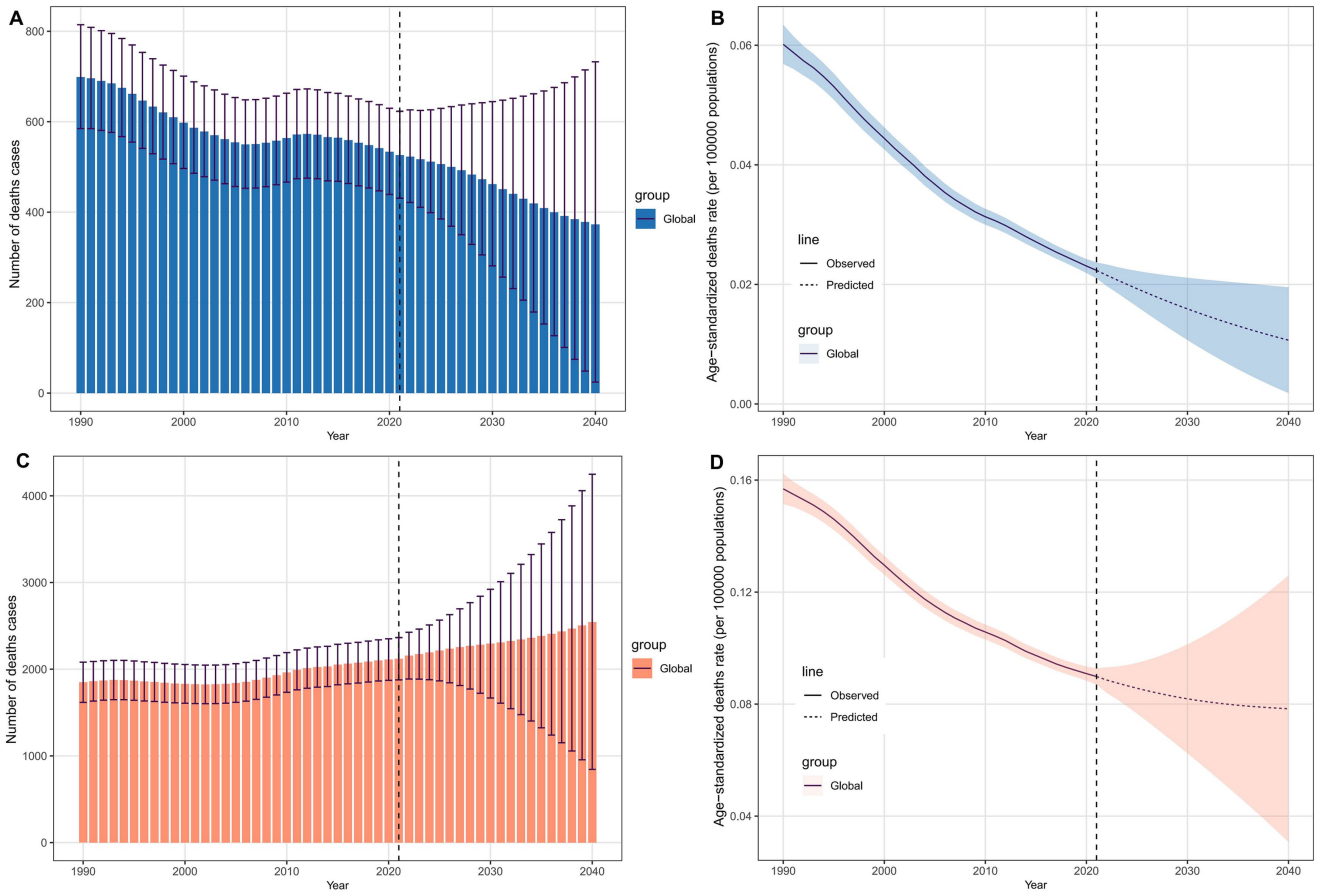
Projected global deaths burden of laryngeal cancer from occupational exposure to 2040. **(A,B)** Asbestos-related deaths and ASDRs. **(C,D)** Sulfuric acid-related deaths and ASDRs.

Figure 7 shows the projected number of deaths and ASDR for LC attributable to occupational exposure by 2040. Globally, it is expected that the number of deaths from LC attributable to occupational exposure to asbestos will decline annually (Figure 7A), reaching 372.78 by 2040. Similarly, the ASDR follows a downward trend, with the rate expected to decrease to 0.011 per 100,000 population by 2040 (Figure 7B). In contrast, for LC attributable to occupational exposure to sulfuric acid, the number of deaths is projected to increase, rising to 2,543.78 by 2040 (Figure 7C), while the ASDR will decrease to 0.078 per 100,000 population (Figure 7D).

### Frontier analysis

**Figure 8:**
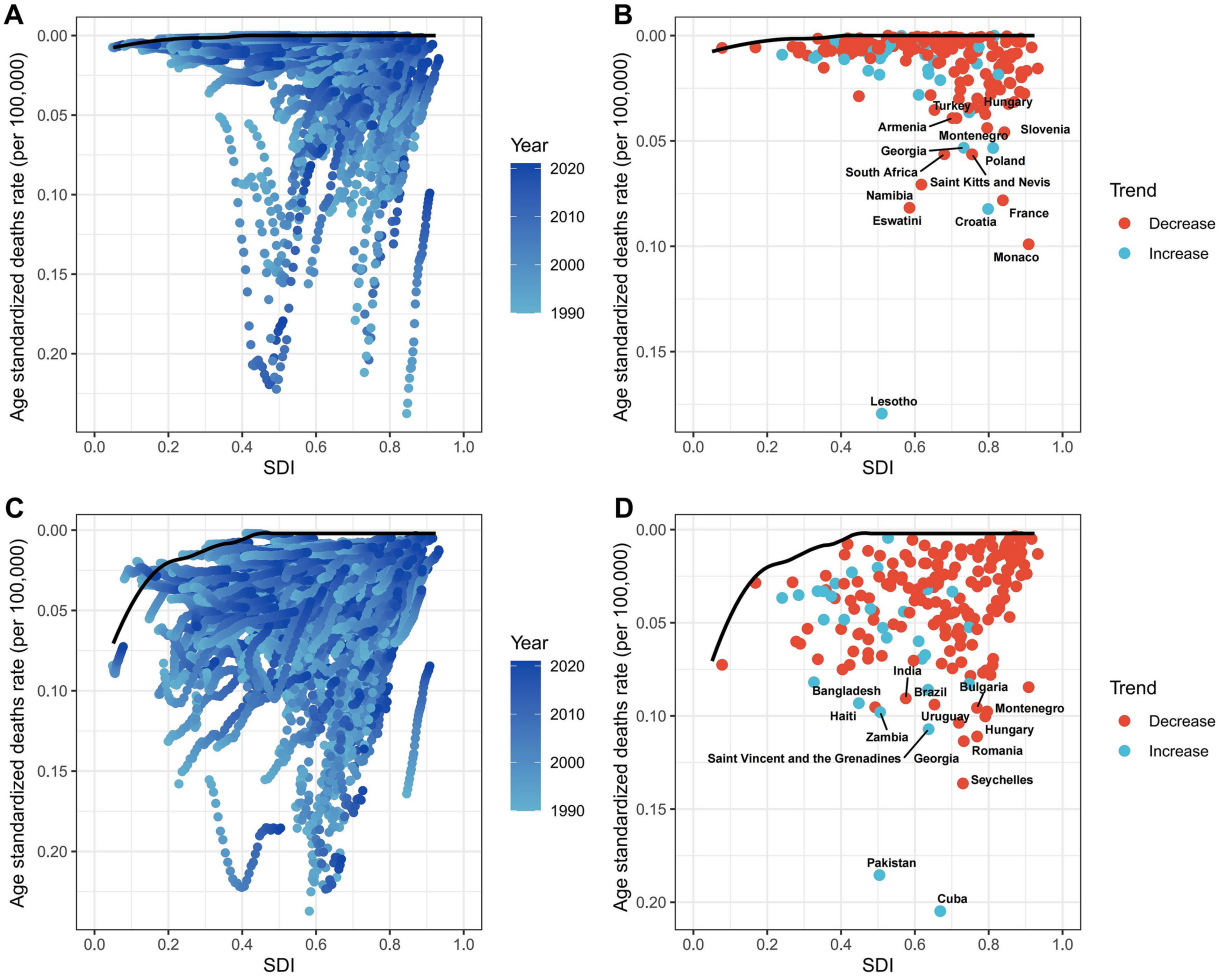
Frontier analysis of SDI and ASDRs in 204 countries, 1990–2021. **(A,B)** Asbestos. **(C,D)** Sulfuric acid.

Using data from 1990 to 2021, we conducted a frontier analysis related to SDI based on the ASDR to explore the potential for reducing the deaths burden of LC attributable to occupational exposure, considering the development levels of countries and regions (Figures 8A,C). The frontier curve represents the minimum (best-performing) ASDR observed for a given SDI, and the distance from the curve, i.e., the effective difference, quantifies the gap between the observed ASDR of a country or region and the achievable ASDR under optimal conditions. In 2021, the effective difference in ASDR for each country was calculated based on their SDI (Figure 8B). Overall, as SDI increases, the effective difference tends to be smaller, with variance stabilizing at higher SDI levels. Regarding occupational exposure to asbestos, in the 15 countries and regions with the greatest potential for improvement, the effective difference ranged from 0.037 to 0.179. These countries and regions include Lesotho, Monaco, Croatia, Eswatini, France, Namibia, Saint Kitts and Nevis, South Africa, Poland, Georgia, Slovenia, Montenegro, Turkey, Armenia, and Hungary (Figure 8B). For occupational exposure to sulfuric acid, the 15 countries and regions with the greatest potential for improvement include Cuba, Pakistan, Seychelles, Georgia, Romania, Saint Vincent and the Grenadines, Uruguay, Hungary, Zambia, Montenegro, Bulgaria, Bangladesh, Brazil, Haiti, and India (Figure 8D).

## Discussion

### Principal findings

This study systematically reveals the global deaths burden of LC attributable to occupational exposure in the male working-age population from 1990 to 2021. The results show that globally, deaths from asbestos-related LC decreased by 22% (with an annual decline of 3.23% in ASDR), while sulfuric acid-related cases increased by 16.19% (with an annual decline of 1.90% in ASDR). High-SDI regions bore a heavier burden from asbestos exposure, while medium-SDI regions faced higher risks from sulfuric acid exposure. Decomposition analysis indicated that the increase in asbestos-related burden was primarily driven by epidemiological factors, while sulfuric acid-related risks were mainly influenced by population growth. By 2040, asbestos-related deaths are projected to decrease to 372 cases (ASDR 0.011/100,000), while sulfuric acid-related deaths are expected to rise to 2,543 cases (ASDR 0.078/100,000), indicating that medium-SDI regions will be a key focus for occupational health prevention and control in the male working-age population.

### Comparison with other studies

Previous study indicated that occupational exposure to carcinogens is a significant cause of death and disability worldwide, and asbestos is a major occupational risk factor. Over the past decades, the burden of occupational cancers has increased significantly, including LC (16). In the 2025 study on the disease burden of LC, three major attributable risk factors for LC were systematically reported, namely smoking, drinking alcohol, and occupational exposure (9). However, previous studies only explored the main attributable risk factors and did not conduct stratified research on the disease burden of each attributable risk factor. Based on previous studies, this research conducted an in-depth investigation into the global disease burden of the two major occupational risk factors for LC among the male labor force. The research on the attributed risk factors of different populations will be conducive to the precise prevention and control of LC. The results of this study show that deaths from asbestos related LC decreased, while sulfuric acid-related cases increased. Therefore, in the future, occupational protection against LC should pay more attention to occupational exposure to sulfuric acid. However, the confidence interval for ASDR is relatively wide. First, the precision of GBD modeling is inherently constrained by data scarcity in low-income regions; occupational exposure misclassification may further bias estimates. Second, while the decomposition model adjusts for smoking and alcohol use, residual confounding persists due to the lack of individual-level data on co-exposures. Third, the assumption of additive independence between demographic and epidemiological factors, though standard in decomposition frameworks, may oversimplify complex interactions in rapidly industrializing settings. These limitations necessitate cautious interpretation of ASDR estimates, particularly in regions with sparse mortality data.

Asbestos is widely used in construction and textiles for its strength, flexibility, heat resistance, and spinnability. Studies show that asbestos fibers, when deposited in the laryngeal mucosa via the respiratory tract, induce chronic inflammation, abnormal cell proliferation, genetic mutations, and, in combination with HPV, increase LC risk by 3.8 times (17). Since 2009, the IARC has classified asbestos as a Group 1 carcinogen for the larynx (18). Numerous studies have highlighted the link between asbestos exposure and LC. A 2020 occupational cancer study reported that asbestos was responsible for 63% of occupational cancer deaths from 2001 to 2016, with males accounting for 79% of these deaths (19, 20). Case–control studies in France and Germany have shown a dose–response relationship between asbestos exposure and LC (21, 22). Given asbestos’ long latency period (average 40 years) and peak use between 1930 and 1970, the peak of asbestos-related LC was expected between 1970 and 2010 (23). Our study shows a 22% decrease in asbestos-related LC deaths in the male working-age population globally from 1990 to 2021, reflecting global asbestos usage trends. The decline in asbestos-related LC deaths is largely attributable to global asbestos restriction policies, heightened occupational awareness, and improved protective measures. Since the International Agency for Research on Cancer (IARC) classified asbestos as a Group 1 carcinogen in 2009, over 60 countries have implemented full or partial bans on its use (18). High-SDI regions demonstrated the most significant ASDR reductions, reflecting stringent workplace regulations and asbestos substitution initiatives (19, 20). Conversely, middle/low-SDI regions with rising asbestos consumption still face increasing risks due to delayed policy implementation and inadequate protective measures. These findings underscore that legislative bans combined with occupational health interventions are effective in reducing asbestos-related cancer burdens, providing a critical roadmap for high-risk nations. Despite increasing recognition of its carcinogenicity, with organizations like ACGIH setting exposure limits, the ILO and WHO emphasize that no safe exposure level exists (24, 25). In recent years, India competes with China to be the largest consumer of asbestos in the world. Other countries among top user are Indonesia and the Russian Federation (10, 26). The import and recycling of asbestos is the chief source in India. In 2016–2017, India imported 310,592 tons of asbestos with 355,686 and 396,493 tons in previous years, respectively (26). Therefore, we call for a comprehensive ban on all types of asbestos worldwide and make the elimination of asbestos related diseases a top priority in the field of public health.

In this study, although high-SDI regions bear a heavier burden from asbestos exposure, decomposition analysis reveals that the primary driver behind this burden in these regions is epidemiological factors, such as improved disease detection. The decline in deaths burden since 1990 in high-SDI regions suggests that prevention and control measures have been effective. In 1983, Iceland became the first country to ban all forms of asbestos nationwide (27). Germany followed suit in 1993, and the use of asbestos in the region is now nearly zero, with over 50 other countries implementing similar bans (28). In recent years, the progress of national asbestos bans has slowed down, with some emerging countries lifting bans and others having long transition periods. This study shows that in low-SDI and some middle-SDI regions, the deaths burden decreased slowly from 1990 to 2021, with some middle-SDI regions even showing a slight increase. In sub-Saharan Southern Africa, the ASDR was significantly higher than expected, indicating insufficient prevention efforts. In middle- and low-SDI countries, the past decades of urbanization and industrialization likely led to extensive asbestos use, and these countries have focused primarily on acute and infectious diseases in their healthcare systems, neglecting sufficient attention to disease prevention and occupational protection.

Frontier analysis indicates that countries with the greatest improvement potential include Lesotho, Monaco, Croatia, Eswatini, France, Namibia, Saint Kitts and Nevis, South Africa, Poland, Georgia, Slovenia, Montenegro, Turkey, Armenia, and Hungary. To further reduce the deaths burden of asbestos-related LC in male working-age populations and eliminate asbestos-related diseases as a public health issue, greater efforts are needed in middle- and low-SDI regions. In countries like Lesotho, where the disease burden exceeds expectations for their development level, governments and public health personnel should conduct further epidemiological investigations to identify targeted prevention measures. Policy and prevention strategies can learn from successful cases in high-SDI regions, such as setting stricter exposure limits, increasing asbestos carcinogenicity education, and replacing asbestos with safer materials to achieve the goal of eliminating asbestos-related diseases at an earlier stage.

Sulfuric acid is widely used in industries such as chemicals, electroplating, and metallurgy, which predominantly involve male workers (7). Inorganic acid mist has been classified as a Group 1 carcinogen since 2012 (29). Although the carcinogenic mechanism of inorganic acid mist is not yet fully understood, low pH is a known pathogenic factor, causing genetic damage and inducing DNA damage in an acidic cellular microenvironment (30). Men, with higher smoking and alcohol consumption rates, may experience an additive effect from sulfuric acid exposure, increasing the risk of LC in the male working-age population. Additionally, higher miR-21 expression in the laryngeal epithelial cells of male patients enhances cell proliferation through the PTEN pathway, and poor adherence to occupational safety further exacerbates the risk (31). Previous studies have shown an increased incidence of LC among acid-pickers and workers in sulfuric acid petrochemical plants, with an exposure-response relationship (31). While cross-sectional and cohort studies at national and subnational levels reflect epidemiological patterns, they cannot fully capture the global and regional disease burden. Research on key global populations will aid in policy formulation and the implementation of prevention and control plans.

This study reveals that from 1990 to 2021, the burden of LC due to occupational sulfuric acid exposure was highest in low- and middle-SDI countries, while high-SDI regions saw a decline, likely due to stricter regulations and better workplace protections (32). Sulfuric acid mist, classified as a Group 1 carcinogen, poses risks primarily through inhalation, especially in poorly ventilated industrial settings. In many developing countries, rapid industrialization, aging populations, and inadequate occupational health systems have led to increasing disease burdens. Informal sectors often lack exposure controls, and regular health screening is limited (18). For example, in China, over 3 million workers in sulfuric acid-related industries have reached high-risk age groups, with aging accounting for over 90% of the disease burden. Countries like Cuba, Pakistan, and the Solomon Islands should strengthen prevention strategies, drawing on best practices from high-SDI nations (31). Targeted efforts are urgently needed in low- and middle-SDI regions to reduce long-term occupational exposure and improve early detection.

By 2040, deaths from occupational sulfuric acid-related LC are expected to rise, although ASDR will decline. For countries like Cuba, with a higher-than-expected burden, public health departments should conduct epidemiological studies to guide prevention measures. In the 15 countries with the greatest improvement potential, targeted prevention and equitable resource distribution are essential. We propose the following actionable measures: (1) low-cost exposure monitoring: Deploy affordable air quality sensors in LMIC workplaces to detect asbestos/sulfuric acid levels, with real-time alerts via SMS. (2) Tax incentives for safer alternatives: Offer 20–30% tax rebates to industries replacing asbestos with cellulose fibers or sulfuric acid with enzymatic cleaners. (3) Primary care task-shifting: Train community health workers to use smartphone-based laryngeal screening tools for early detection in high-risk groups. (4) Policy integration: Embed occupational exposure limits into existing national NCD prevention programs.

### Strengths and limitations

This study maps the global spatiotemporal characteristics of occupational sulfuric acid and asbestos-related LC in male working-age populations, filling a gap in occupational cancer epidemiology. By using decomposition models, it identifies 15 countries, such as Lesotho (asbestos) and Cuba (sulfuric acid), as key targets for intervention. However, limitations include: (1) data limitations due to underreporting in low-income countries; (2) historical bias as the study covers only 1990–2021 data, missing earlier high-exposure populations; (3) confounding factors, such as smoking and alcohol, not fully controlled for; (4) binary exposure classification, which cannot assess exposure gradients; (5) lack of exposure scenario differentiation, potentially missing key risk contexts. Future research should use multicenter cohort studies and dynamic policy models to improve burden assessments.

## Conclusion

The LC attributable to occupational exposure in male working-age populations shows significant divergence: asbestos-related deaths continue to decrease, while sulfuric acid-related cases rise annually. High-SDI regions still face a substantial asbestos burden due to historical factors, while rapidly industrializing middle- and low-SDI regions experience increasing sulfuric acid exposure risks. Asbestos harm is mainly driven by delayed carcinogenic effects, while sulfuric acid risk is closely linked to population growth and aging. Future prevention and control should be targeted: high-SDI regions should focus on managing asbestos legacy risks, while middle- and low-SDI regions need stronger industrial safety systems. Global collaboration to promote alternative materials and screening mechanisms is essential for reducing disease burdens equitably. Continued research into carcinogenic mechanisms and optimized prevention strategies remains crucial.

## Data Availability

The datasets presented in this study can be found in online repositories. The names of the repository/repositories and accession number(s) can be found: all data used in this study can be freely accessed at the Global Health Data Exchange (GHDx) query tool (https://vizhub.healthdata.org/gbd-results/).

## Footnotes

^1^ https://vizhub.healthdata.org/gbd-results/

^2^ https://ghdx.healthdata.org/gbd-2021
